# A Medium-Frequency Fiber Bragg Grating Accelerometer Based on Flexible Hinges

**DOI:** 10.3390/s21216968

**Published:** 2021-10-20

**Authors:** Zichuang Li, Lei Liang, Hui Wang, Shu Dai, Ke Jiang, Zhiyuan Song

**Affiliations:** National Engineering Laboratory for Fiber Optic Sensing Technology, Wuhan University of Technology, Wuhan 430070, China; lizichuang@whut.edu.cn (Z.L.); wanghui1989@whut.edu.cn (H.W.); 290303@whut.edu.cn (S.D.); k.jiang@whut.edu.cn (K.J.); 291875@whut.edu.cn (Z.S.)

**Keywords:** fiber Bragg grating, accelerometer, flexible hinge, vibration monitoring

## Abstract

Mediumfrequency fiber Bragg grating (FBG) acceleration sensors are used in important applications in mechanical, aerospace and weapon equipment, and have strict requirements in terms of resonance frequency and sensitivity. A novel medium-frequency accelerometer, based on fiber Bragg grating and flexible hinges, is proposed in this paper. The differential structure doubles the sensitivity of the sensor while avoiding temperature effects. The structure model and principle for the sensor are introduced, the sensor’s sensing characteristics are theoretically analyzed, and the structure parameters for the sensor are determined through numerical analysis. The sensing experiments show that the resonance frequency of the sensor is approximately 2800 Hz, the sensitivity is 21.8 pm/g in the flat frequency range of 50–1000 Hz, and the proposed sensor has a good temperature self-compensation function and lateral anti-interference capability.

## 1. Introduction

Electronic acceleration sensor technology is relatively mature, and it is widely used in the field of mechanical fault diagnosis [1,2,3]. Compared with traditional electronic sensors, FBG sensors have many advantages, including anti-electromagnetic interference, corrosion resistance, long-distance transmission, and distributed measurement. FBG sensors are widely used in important fields, such as railways [4], bridges [5], the oil industry [6], and aerospace [7,8]. Up until the present day, FBG acceleration sensors have been widely used in vibration measurement, playing an important role in structural health monitoring [9,10,11,12,13]. A packaged FBG acceleration sensor is composed of a spring-mass system with one or several FBGs. Various elastic elements have been studied to form FBG acceleration sensors, including cantilever type [14,15,16,17,18], diaphragm type [19,20,21], and flexure hinge type [22,23,24,25,26,27]. In flexure hingebased acceleration sensors [23,24,25], the two sides of a flexure hinge are connected with the mass and the base, respectively, and the FBG is placed between the base and the inertial mass to realize the acceleration measurement. Wei et al. [26] arranged two couple FBGs to eliminate the influence of temperature and double the sensitivity of the sensor. The acceleration sensitivity reached 40 pm/g, but its resonance frequency is as low as 814.3 Hz. Wang et al. [27] realized the measurement of three-dimensional acceleration based on a composite flexible hinge. The sensor’s *z*-axis sensitivity is 20.3 pm/g, and the measurement frequency range is only 0–250 Hz, which is unable to measure medium–high-frequency acceleration.

In recent years, many scholars proposed various structures for medium–high-frequency FBG acceleration sensors [28,29,30,31,32]. In [28], Stefani et al. proposed an FBG accelerometer based on polymer fiber and a flexible hinge structure. The sensor’s resonance frequency is approximately 3 kHz, and the sensitivity is up to 19 pm/g. The study pointed out that polymer FBGs can increase the sensitivity of an accelerometer by more than four times, compared with silica FBGs. In [29], based on the flexible hinge structure, Dai et al. discussed the influence of hinge structure parameters on the sensor’s sensitivity and resonance frequency, and reported an accelerometer with a resonance frequency of about 3000 Hz. Their experiments showed that the sensor sensitivity is 13.82 pm/g at an excitation frequency of 100 Hz. In [30], Guo et al. introduced a welding-packaged accelerometer based on a metal-coated FBG; the resonance frequency and sensitivity of the sensor are 3.6 kHz and 1.7 pm/g, respectively. In [31], based on the elastic structure of a steel tube and a mass block, Wang et al. designed a high-frequency FBG accelerometer. Embedded with two FBGs for axial vibration sensing, the resonance frequency and sensitivity are 3806 Hz and 4.01 pm/g, respectively. According to [32], Wu et al. reported a high-frequency FBG accelerometer with an isosceles triangular cantilever beam and a mass block. In this sensor, one FBG is pasted onto the cantilever beam for measurement and the other FBG is used as a reference. Adopting light intensity demodulation, the resonance frequency and sensitivity of the sensor are 8193 Hz and 0.46 pm/g, respectively.

For FBG acceleration sensors, the resonance frequency and sensitivity restrict each other, which is also mentioned in some related studies [21]. In existing reports about mediumhigh frequency FBG acceleration sensors, the sensitivities are often low, which leads to low resolution. A possible way to improve the sensor performance is to use FBGs in polymer optical fiber, but polymer optical fiber will bring higher transmission losses. Therefore, we try to broaden the frequency measurement range and increase the sensitivity by improving the structure of the sensor.

In this work, we propose a novel medium-frequency FBG accelerometer based on two FBGs, three flexible hinges, and two inertial mass blocks, where the three straight circular flexure hinges connect the two masses and the base into a whole. Two FBGs are then arranged symmetrically at both ends of the two masses. Such a hingemasshinge structure can improve the overall rigidity of the sensor compared with the common masshingebase structure. Meanwhile, two FBGs suspended between two masses not only doubles the sensitivity of the sensor, but also leads to a temperature self-compensation capability. In the following sections, the sensing characteristics of the sensor are analyzed theoretically, the structural parameters are determined by numerical analysis, and, finally, the sensor is experimentally studied.

## 2. Materials and Methods

### 2.1. Structure Model and Principle of the Sensor

**Figure 1:**
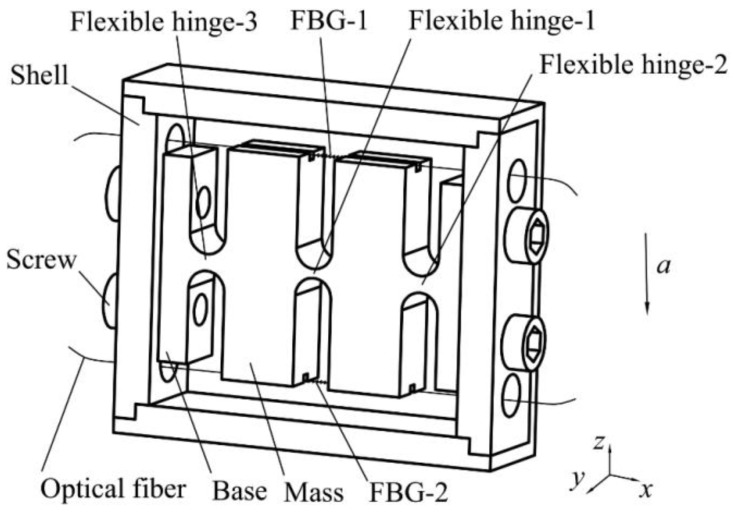
Structural diagram of the FBG accelerometer.

The detailed design of the proposed medium-frequency FBG accelerometer is illustrated in Figure 1. This sensor is mainly composed of two inertial masses, three straight circular flexure hinges, two FBGs and a base. The sensor is symmetrical, the sizes of flexure hinge-2 and flexure hinge-3 are exactly the same. The two ends of the masses are provided with optical fiber grooves. The two ends of the pre-stretched FBG are fixed in the grooves by glue. The FBG is suspended above the flexible hinge, and the base and the package shell are connected by threads.

When an acceleration excitation along the measurement direction is generated externally, the two masses will rotate slightly around the center of flexible hinge-2 and flexible hinge-3, respectively, driving the FBGs to stretch or compress and converting the vibration acceleration into the strains and wavelength shifts of two FBGs. Vibration signals can be obtained from the wavelength shifts of the two FBGs. In this design, three straight round flexible hinges connect the two masses and the base into a whole. The hinge–mass–hinge structure can improve the overall rigidity of the sensor compared with the hinge-mass structure. Meanwhile, two FBGs designs double the sensor’s sensitivity while realizing temperature self-compensation.

### 2.2. Sensitivity

**Figure 2:**
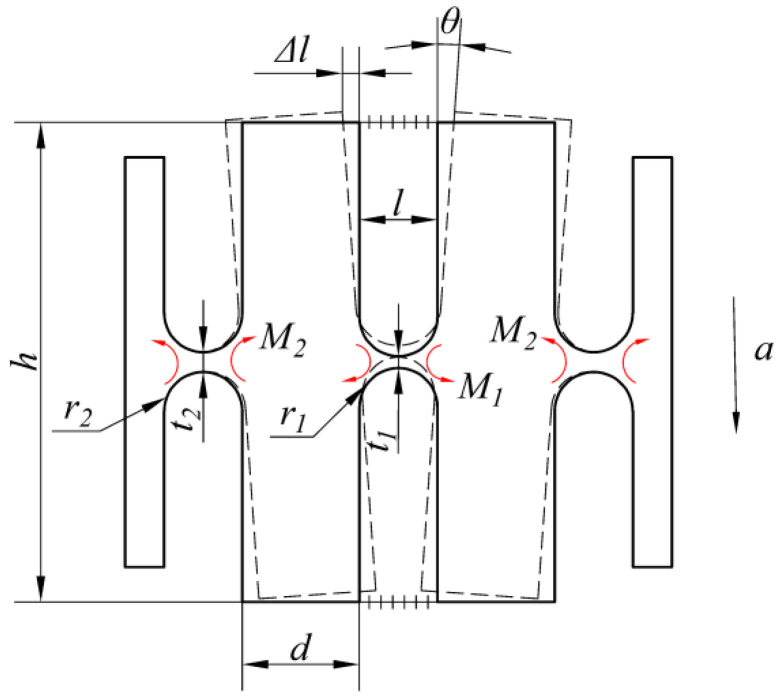
Mechanical model of the FBG accelerometer.

When an external acceleration acts on the sensitive direction of the sensor, the two masses will rotate slightly around the center of flexible hinge-2 and flexible hinge-3, respectively, under the action of inertial force. The mechanical model of the sensor is shown in Figure 2. 

According to the principle of virtual displacement, the general dynamics equation for the system can be expressed as follows:

(1) $$(-2M_{1}-2M_{2})\delta \theta +2ma\delta z-4F_{f}\delta \Delta l=0$$

where *θ* is the rotation angle of the mass relative to flexible hinge-2, *m* is the mass of the mass block, *a* is the vibration acceleration in the *z*-axis of the sensor, *z* is the displacement of the mass center of the mass in the *z*-axis, *F_f_* is the tensile force generated by the optical fiber, *l* is the optical fiber pasting span, and Δ*l* is the displacement of the bonding point at one end of the optical fiber in the *x*-axis.

The magnitude of the moment *M_i_* is proportional to the stiffness *K_i_* of the straight circular flexure hinge, which can be expressed as follows:

(2) $$M_{i}=K_{i}\theta \;(i=1,2)$$

Here, the stiffness of the straight round flexure hinge is given by [33].

(3) $$K=\frac{{\mathrm{EwR}}^{2}}{24}/\left[\frac{s^{3}\left(6s^{2}+4s+1\right)}{\left(2s+1\right){\left(4s+1\right)}^{2}}+\frac{6s^{4}\left(2s+1\right)}{{\left(4s+1\right)}^{5/2}}\arctan\sqrt{4s+1}\right]$$

(4) $$s=\frac{r}{t}$$

where *E* is the elastic modulus of the flexible hinge, *r* is the radius of the straight circular flexible hinge, *t* is the thickness of the hinge waist, and *w* is the width of the hinge.

In Equation (1), the magnitude of the pulling force can be obtained by the following:

(5) $$F_{f}=2k_{f}\Delta l$$

where the following is the elastic coefficient of the optical fiber:

(6) $$k_{f}=\frac{A_{f}E_{f}}{l}$$

where *l* is the bonding span of the optical fiber, and *A_f_* and *E_f_* are the cross-sectional area and elastic modulus of the optical fiber, respectively.

Since *θ* is very small, we can take sin*θ* ≈ *θ*. From the geometric relationship, we have the following:

(7) $$z=(r_{2}+\frac{d}{2})\theta$$

(8) $$\Delta l=\frac{h}{2}\theta$$

When deformation occurs between spans, the strain ε corresponding to the grating can be described as follows:

(9) $$\varepsilon =\frac{2\Delta l}{l}$$

When the incident light passes through the FBG, part of light that meets the Bragg central wavelength condition will be reflected. External strain and temperature load will result in a shift of the Bragg central wavelength. The effects of strain and temperature on the Bragg wavelength shift can be expressed as follows:

(10) $$\frac{\Delta \lambda }{\lambda }=\left(1-P_{e}\right)\varepsilon +\left(\alpha_{f}+\xi_{f}\right)\Delta T$$

where *λ* is the Bragg central wavelength for the FBG, Δ*λ* is the Bragg wavelength shift of the FBG, α_f_ is the thermal expansion coefficient, ζ_f_ is the thermo-optical coefficient, and *P*_e_ is the photoelastic coefficient (theoretical value = 0.22).

For FBG-1 and FBG-2, the following apply:

(11) $$\frac{\Delta \lambda_{1}}{\lambda_{1}}=\left(1-P_{e}\right)\varepsilon_{1}+\left(\alpha_{f}+\xi_{f}\right)\Delta T$$

(12) $$\frac{\Delta \lambda_{2}}{\lambda_{2}}=\left(1-P_{e}\right)\varepsilon_{2}+\left(\alpha_{f}+\xi_{f}\right)\Delta T$$

where *λ*_1_ and *λ*_2_ represent the central wavelengths for FBG-1 and FBG-2 after prestretching, Δ*λ*_1_ and Δ*λ*_2_ represent the central wavelength shifts for FBG-1 and FBG-2 after prestretching, respectively, and *ε*_1_ and *ε*_2_ represent the strain amounts for FBG-1 and FBG-2 after prestretching, respectively.

FBG-1 and FBG-2 are symmetrically arranged at both ends of the mass block. When FBG-1 is stretched, FBG-2 is compressed, with *ε*_1_ = *ε* and *ε*_2_ = −*ε*. The temperature sensitivity coefficients for the selected gratings are the same, and the central wavelengths are approximately equal, with *λ*_1_ = *λ*_2_ = *λ*.

By combining Equations (11) and (12) to eliminate the influence of temperature on the wavelength shift of the grating, we can obtain the following:

(13) $$\Delta \lambda =\Delta \lambda_{1}-\Delta \lambda_{2}=2\left(1-P_{e}\right)\varepsilon$$

Combining Equations (1), (7), (8) and (13), the sensitivity of the sensor can be expressed as follows:

(14) $$S=\frac{\Delta \lambda }{a}=\frac{4\lambda (1-P_{e})\Delta l}{al}=\frac{\lambda (1-P_{e})}{l}\frac{2mh(r_{2}+\frac{d}{2})}{k_{f}h^{2}+K_{1}+K_{2}}$$

### 2.3. Resonance Frequency

The resonance frequency is an important indicator to account for the performance of an accelerometer. Suppose *J* is the inertia moment of the mass around the center of hinge-2 and *θ* is the generalized coordinate for obtaining the Lagrangian function, as follows:

(15) $$L=T_{m}-V_{f}-V_{J}$$

The strain potential energy of the optical fiber can be expressed as follows:

(16) $$V_{f}=2\times \frac{1}{2}k_{f}{\left(h\theta \right)}^{2}$$

The elastic potential energy of the hinge can be obtained by the following:

(17) $$V_{J}=2\times \frac{1}{2}(K_{1}+K_{2})\theta^{2}$$

The kinetic energy of the mass block can be described as follows:

(18) $$T_{m}=2\times \frac{1}{2}J{\dot{\theta }}^{2}$$

The Lagrangian equation for the conservative force can be written as follows:

(19) $$\frac{\partial }{\partial t}(\frac{\partial L}{\partial \dot{\theta }})-\frac{\partial L}{\partial \theta }=0$$

Substituting Equation (15) to (18) into Equation (19), the dynamic equation for the system can be described as follows:

(20) $$2J\ddot{\theta }+(2k_{f}h^{2}+2(K_{1}+K_{2}))\theta =0$$

The resonance frequency of the system can be expressed as follows:

(21) $$f=\frac{1}{2\pi }\sqrt{\frac{k_{f}h^{2}+(K_{1}+K_{2})}{J}}$$

According to the moment of inertia formula and the parallel axis theorem, the moment of inertia can be obtained by the following:

(22) $$J=m\frac{d^{2}+h^{2}}{12}+m(r_{2}+\frac{d}{2}{)}^{2}$$

### 2.4. Dimensional Parameter Optimization

Since the sensor is symmetrical, only the influence of flexible hinge-1 and flexible hinge-2 on the resonance frequency and sensitivity of the sensor is discussed. It can be seen from Equations (14) and (21) that the radius (*r*_1_) and waist thickness (*t*_1_) of hinge-1, the radius (*r*_2_) and the waist thickness (*t*_2_) of hinge-2, the thickness of hinge (*w*), and the width (*d*) and height (*h*) of the mass block are seven key parameters that affect the resonance frequency and sensitivity of the sensor. To obtain a higher resonance frequency and greater sensitivity, the structural parameters of the sensor need to be optimized.

Since the length of the FBG grating used is 5 mm, to increase the sensitivity, the radius *r*_1_ is set to 2.5 mm. To ensure reliable fixation at both ends of the optical fiber, the width of mass block *d* is set to 10 mm. To limit the sizes of the sensor, the height *h* is set to 30 mm, and the thickness *w* is 8 mm. The sensor adopts wire-cutting integrated processing. The material characteristic parameters are shown in Table 1.

**Figure 3:**
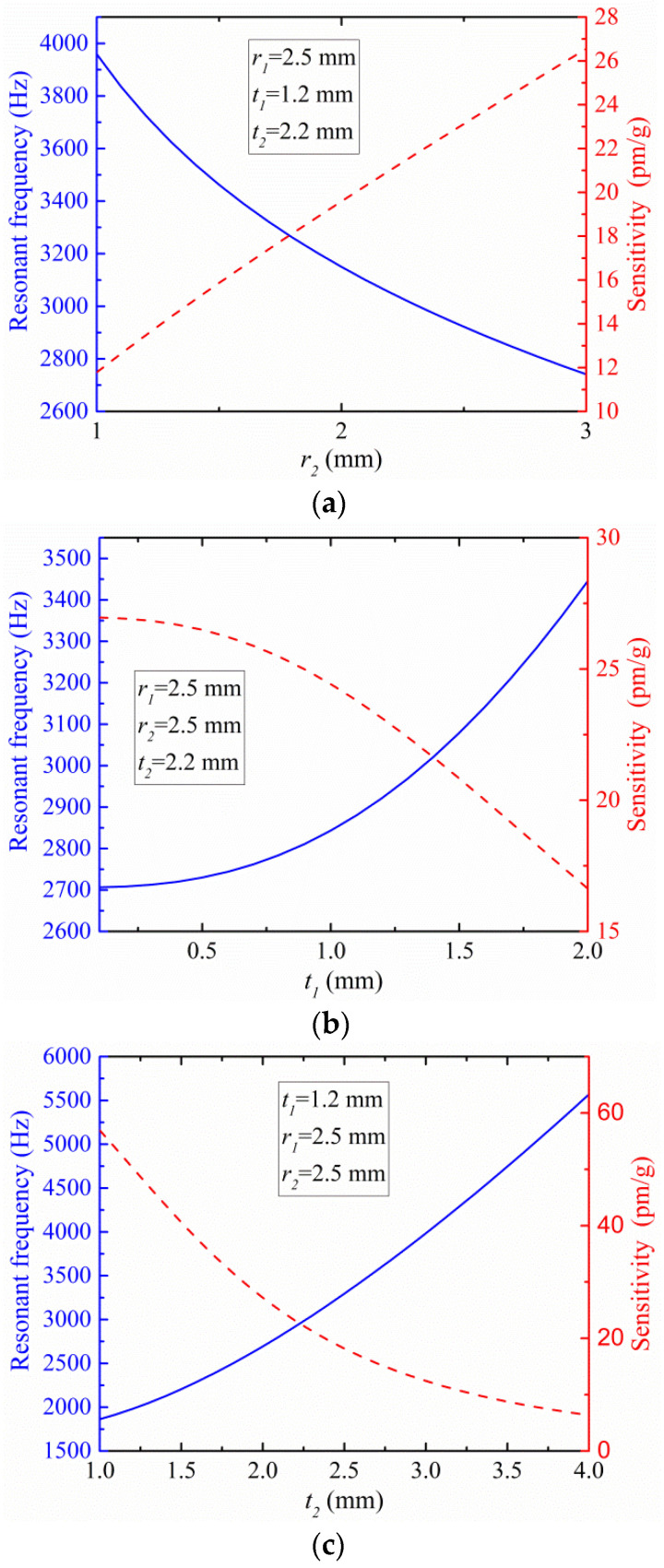
Dependence relationship of the resonance frequency and sensitivity on the (**a**) radius of hinge-2 (*r*_2_), (**b**) waist thickness of hinge-1 (*t*_1_), and (**c**) waist thickness of hinge-2 (*t*_2_).

So, we have investigated the influence of *r*_2_, *t*_1_, and *t*_2_ on the sensor’s resonance frequency and sensitivity to determine the optimal size parameters. The dependence relationships of the resonance frequency and sensitivity on three dimensional parameters (*r*_2_, *t*_1_, *t*_2_) are shown in Figure 3a–c.

As shown in Figure 3a, the resonance frequency decreases with increasing radius of flexible hinge-2. When 1 mm ≤ *r*_2_ ≤ 3 mm, the resonance frequency has a larger variation range, which can be used to adjust the working frequency range of the sensor. Compared with *t*_1_ and *t*_2_, *r*_2_ has a greater impact on the size of the sensor, and *r*_2_ should not be too large.

As shown in Figure 3b, the resonance frequency increases as the thickness of the thinnest part of flexure hinge-1 *t*_1_ increases. When 0.5 mm ≤ *t*_1_ ≤ 2 mm, the resonance frequency changes in a smaller range. 

As shown in Figure 3c, the resonance frequency increases as the thickness of the thinnest part of flexure hinge-2 *t*_2_ increases. When 1 mm ≤ *t*_2_ ≤ 4 mm, the resonance frequency shows a large variation range, which can be used to adjust the operating frequency range. In addition, *t*_2_ should not be too small, and sufficient connection strength between the mass and the base should be maintained. When 1 mm ≤ *t*_2_ ≤ 2.5 mm, the sensitivity range is also very large, and the mutual restriction of sensitivity and resonance frequency is obvious. To obtain a higher resonance frequency and greater sensitivity, 2 mm ≤ *t*_2_ ≤ 2.5 mm was selected.

This paper aims to design a medium-frequency accelerometer with a resonance frequency of about 3000 Hz. While ensuring that the sensor obtains a higher resonance frequency and greater sensitivity, we determined the structure size parameters. The structure and material characteristics of the FBG accelerometer are shown in Table 1. Based on the data in Table 1, the theoretical resonance frequency and sensitivity of the sensor are 2922.1 Hz and 23.1 pm/g, respectively.

## 3. Simulation Analysis of the Sensor

To further study the dynamic characteristics of the sensor, ANSYS software was used to perform analysis for the sensor model. According to the size parameters given in Table 1, a three-dimensional model of the sensor was established through SOILDWORKS software and imported into ANSYS software. The material properties of the model were set according to the material characteristic parameters in Table 1. The hexdominant grid division was complete, with the grid element size being equal to 0.3 mm. Fixed support constraints were imposed on the bases at both ends of the sensor. The optical fiber was modeled by spring constraints with the same elastic coefficient, suspended above and below flexible hinge-1.

### 3.1. Model Analysis

**Figure 4:**
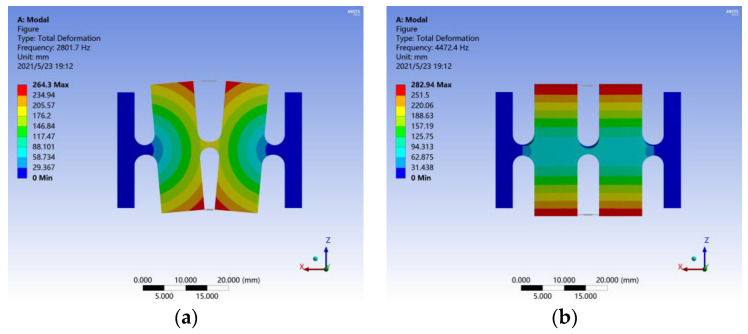
Model analysis results for the FBG accelerometer: (**a**) first-order and (**b**) second-order.

The model was solved to obtain the sensor’s deformation cloud diagram. As shown in Figure 4a, the first-order model shape involves two masses rotating slightly around the center of the flexible hinge connected to the base. The first-order resonance frequency is 2801.7 Hz, which is quite consistent with the theoretical value (2922.1 Hz). As shown in Figure 4b, the second-order model shape of the sensor involves a lateral vibration of two masses along the *y*-axis, and the second-order resonance frequency is 4472.4 Hz. These data show that the resonance frequency of the lateral vibration is 59% higher than the main resonance frequency, indicating that the sensor has a strong ability to resist lateral interference.

### 3.2. Harmonic Response Analysis

**Figure 5:**
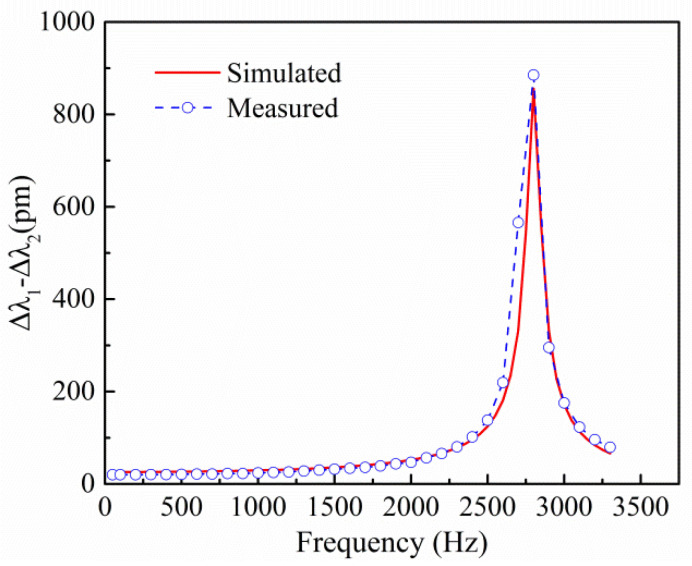
Amplitude-frequency response curves for the sensor.

An excitation acceleration of 1 g was applied along the *z*-axis of the sensor. The constant damping ratio was set to 0.015. The excitation frequency varied in the range from 50 Hz to 3300 Hz, with a step length of 50 Hz. The maximum displacements of the fiber’s fixed points on top of the masses were calculated and converted into two FBG wavelength shifts (Δ*λ*_1_–Δ*λ*_2_). The simulated curve in Figure 5 shows the harmonic response of the sensor, and the first-order resonance frequency is approximately 2800 Hz, which is basically consistent with the theoretical value (2922.1 Hz). At an excitation signal frequency of 50 Hz, the sensor’s sensitivity is 25.7 pm/g, which is basically consistent with the theoretical value (23.1 pm/g).

## 4. Experimental Characterization of the Sensing Properties

### 4.1. Fabrication of Sensor

According to the sensor’s structure shown in Figure 1 and the parameters shown in Table 1, the sensor was directly processed into a one-piece structure by a piece of 304 stainless steel, using the slow wire electrical discharge machining (EDM) method. The machining surface of the slow wire EDM method is smooth, and the machining accuracy is high. Two FBGs (silica fiber with an outer diameter of 125 μm, Shenzhen AtGrating., Ltd., Shenzhen, China; 1545.785 nm, 1546.025 nm; 5 mm length, >70% reflectivity) were used. Adhesive 353ND (Epoxy Technology, USA) is a high-temperature-resistant epoxy designed for semiconductor, hybrid, optical fiber and medical applications. To obtain a better paste effect, firstly, about 5 mm of coating was removed at the two sides of the FBG before pasting. Then, the peeled parts were placed on the optical fiber grooves tightly. At the same time, pre-tension was applied to the FBG by hanging a weight of 300 g. When the temperature increased to 120 °C, 353ND epoxy adhesive was applied evenly to the peeled parts on both sides of the FBG, and curing was achieved by heating the epoxy at 120 °C for 15 min. After fabrication, annealing was carried out at 80 °C in an oven before the experiment.

### 4.2. Experimental System Compositions

**Figure 6:**
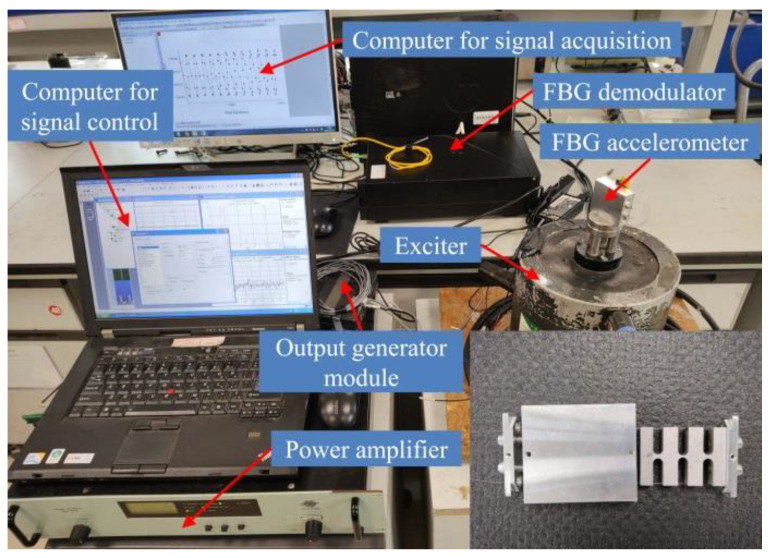
FBG accelerometer experimental system.

The experimental system for the FBG accelerometer is shown in Figure 6. The experimental system consists of the following two parts: a vibration sensing system and a demodulation system. The vibration sensing system (LAN-XI, made by the B&K Company, Copenhagen, Denmark) includes an FBG accelerometer, a vibration exciter, a power amplifier, a standard reference acceleration sensor, and a computer. The computer is responsible for controlling the frequency and amplitude of the vibration exciter. The demodulation system consists of the FBG demodulator and signal acquisition software. The FBG demodulator was self-developed, based on an FPGA-IRS demodulation module produced by BaySpec Inc. The maximum sampling frequency was 8 kHz, and the resolution was 1 pm. The demodulation system is responsible for collecting the central wavelength signal of the reflected light from the sensor grating. The proposed accelerometer was fixed onto the vibration axis of the vibration exciter, and the two FBGs in this accelerometer were connected to the demodulator through an optical fiber. The experiment was carried out at room temperature (25 °C).

### 4.3. Amplitude-Frequency Response

To study the amplitude-frequency characteristics of the sensor, the amplitude of the excitation acceleration was kept at 1 g, and the excitation frequency was varied in the range of 50 Hz to 3300 Hz, with a step of 100 Hz. The relationship between the central wavelength difference (Δ*λ*_1_–Δ*λ*_2_) of the two FBGs and the frequency under different excitation frequencies was measured, and the amplitude-frequency response curve is shown in Figure 5. The response curve shows that the resonance frequency of the accelerometer is approximately 2800 Hz, which is basically consistent with the theoretical value (2922.1 Hz) and the finite element simulation data (2801.7 Hz). The amplitude-frequency curve has better flatness below 1000 Hz, and the operating frequency range of the sensor is 50–1000 Hz.

### 4.4. Sensitivity

**Figure 7:**
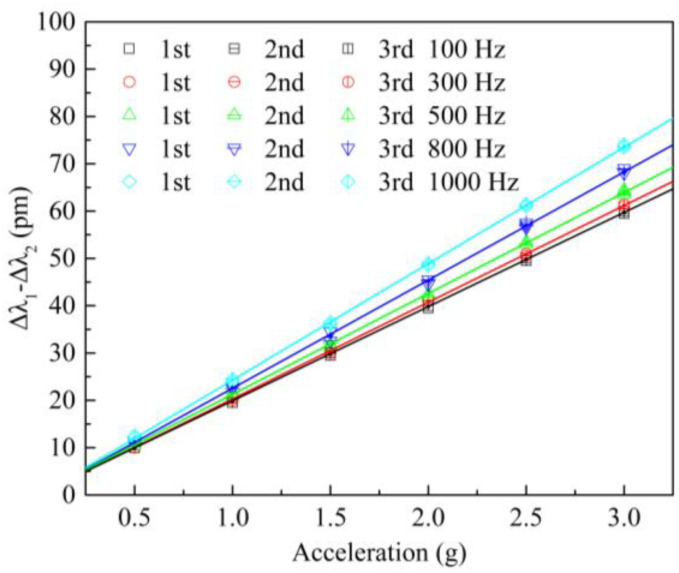
Wavelength shift difference versus acceleration amplitude at different excitation frequencies.

In the sensitivity experiment, since the sensor adopts a dual FBG differential structure, the wavelength shift difference (Δ*λ*_1_–Δ*λ*_2_) for the two FBGs was selected as the output response. Through the vibration exciter, sinusoidal excitation signals of 100 Hz, 300 Hz, 500 Hz, 800 Hz, and 1000 Hz were given to the sensor, and the acceleration amplitude increased from 0.5 g to 3 g, with a step length of 0.5 g. Each group of experiments was repeated three times. The variations for the wavelength shift difference, with acceleration at different frequencies, are shown in Figure 7—by open square, circles, up-triangles, down-triangles, and diamonds. 

The average values of the three sets of experimental data at different frequencies are denoted by solid circles. Progressively, for the average values at each frequency, the least-square fitting method was used to acquire its linear fitting line, which was denoted by solid lines (black, red, green, blue, and cyan). At excitation frequencies of 100 Hz, 300 Hz, 500 Hz, 800 Hz, and 1000 Hz, their slopes, meaning the sensitivities of the sensor, are 19.9 pm/g, 20.4 pm/g, 21.3 pm/g, 22.8 pm/g, and 24.6 pm/g, respectively. These experimental data show that the wavelength shift difference for the sensor has a good linear relationship with the acceleration amplitude. Within the working frequency of 50–1000 Hz, the sensor obtained an average sensitivity of 21.8 pm/g. The main reasons for the difference between theoretical sensitivity and experimental sensitivity are dimensional deviations in the sensor, in its slow wire EDM and packaging.

The Bessel formula was used to calculate the standard deviation. First, all the subsamples’ standard deviations *σ_i_* were calculated, where *i* = 1, 2 … *N*, and *N* = 6 is the number of calibration points. Then, the standard deviation of the sensor was calculated using $\sigma =\sqrt{\frac{1}{N}\sum_{i=1}^{N}\sigma_{i}^{2}}$. Finally, the repeatability error was calculated as *e_R_* = *ησ*/*λ*_FS_, where *η* = 3, which is the coverage factor, and *λ*_FS_ is the maximum wavelength shift. As shown in Figure 7, when the frequency of the excitation signal is 100 Hz, 300 Hz, 500 Hz, 800 Hz, and 1000 Hz, the repeatability errors for the sensor are 2.7%, 2.4%, 2.7%, 3.3%, and 2.2%, respectively.

### 4.5. Temperature Self-Compensation

**Figure 8:**
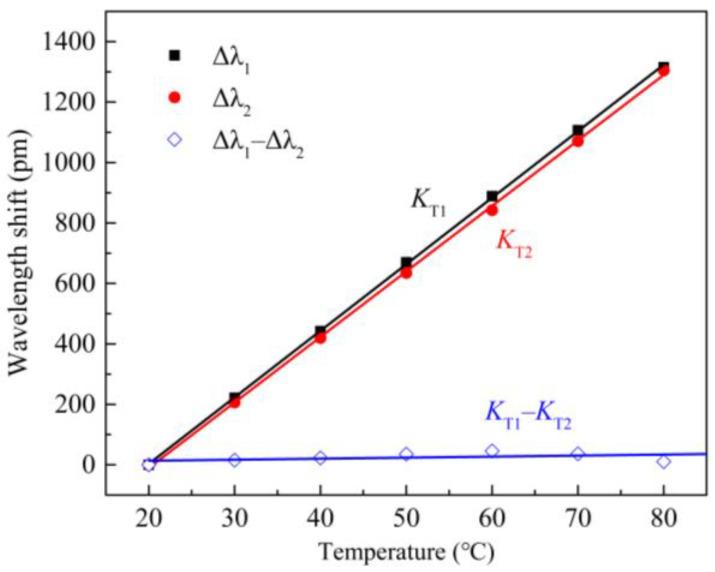
Temperature responses and self-compensation for the FBG accelerometer.

Since FBGs are sensitive to both strain and temperature, to study the temperature self-compensation performance of the sensor, a temperature control box (SDEI, SDJ402F, adjustment range of 10–200 °C, resolution of 0.01 °C, measurement accuracy of 0.05 °C) was used to test the sensor. The sensor was placed in the temperature control box, and the temperature was adjusted from 20 °C to 80 °C, in steps of 10 °C. The central wavelength shift was measured for the two FBGs at different temperatures, and the temperature response curves obtained for Δ*λ*_1_, Δ*λ*_2_, and Δ*λ*_1_–Δ*λ*_2_ are shown in Figure 8. The temperature sensitivity coefficients for FBG-1 and FBG-2 are *K*_T1_ = 22.01 pm/°C and *K*_T2_ = 21.67 pm/°C, respectively, and their linear correlation coefficients are 0.999. The experimental results show that the sensitivity coefficients for the two FBGs are basically the same, and the sensor has good temperature self-compensation capability.

### 4.6. Cross-Interference Characteristic

**Figure 9:**
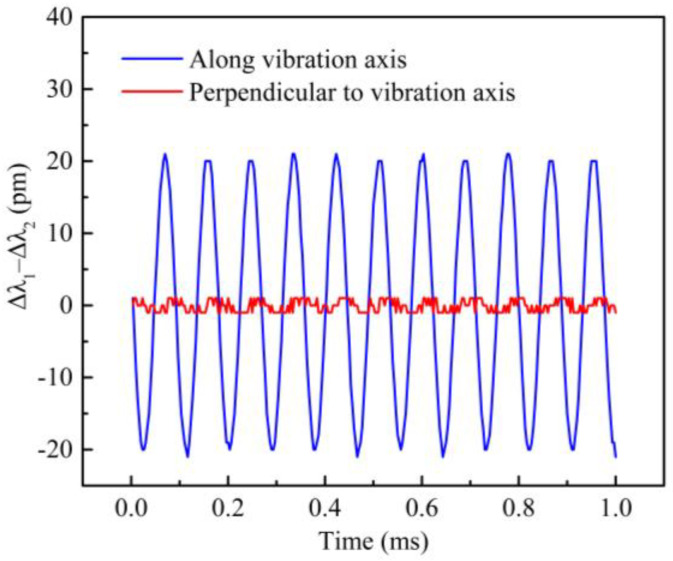
Wavelength shift difference of the sensor when the vibration is along or perpendicular to the vibration axis of exciter.

Anti-interference ability is also an important indicator for accelerometers, so an anti-interference experiment was also finished, where the sensor was fixed onto the vibration exciter along the main axis of the vibration exciter and in its vertical direction, respectively. A sinusoidal excitation signal, with frequency of 300 Hz and amplitude of 1 g, was applied to the sensor. The wavelength shift differences of the sensor were obtained in the main and lateral directions, as shown in Figure 9. The peak–peak value of the central wavelength difference for the sensing in the main direction is about 41 pm, but the peak–peak value in the lateral direction does not exceed 2 pm; therefore, the lateral interference degree for the sensor is less than 5%, which shows that the designed sensor has a good anti-interference ability.

Table 2 gives a characteristics comparison of the proposed medium–high-frequency FBG accelerometer with other FBG accelerometers, based on different structures, including resonance frequency, sensitivity, FBG type, and temperature self-compensation capabilities.

## 5. Conclusions

A novel medium-frequency FBG accelerometer, based on two FBGs, three flexible hinges, and two inertial mass blocks, was designed and prepared. Theoretical analysis and finite element simulation were used to determine the optimal parameters of the sensor. The sensing experiments show that the proposed sensor had a resonance frequency of about 2800 Hz and a sensitivity of 21.8 pm/g in the flat frequency range of 50–1000 Hz. The sensor’s resonance frequency and sensitivity, given by theoretical analysis, finite element simulation, and sensing experiments, are basically consistent. In addition, the experiments also show that the sensor has a good temperature self-compensation function (temperature sensitivity *K*_T1_–*K*_T2_ ≈ 0.34 pm/°C) and lateral anti-interference capability (lateral interference degree is less than 5%). These performances provide a novel and reliable method for the engineering application of medium-frequency FBG acceleration sensors.

## Figures and Tables

**Table 1:** Parameters for the FBG accelerometer structure and material properties.

Parameter	Parameter Description	Value (Units)
*r*_1_	Radius of hinge-1	2.5 mm
*t*_1_	Waist thickness of hinge-1	1.2 mm
*r*_2_	Radius of hinge-2	2.5 mm
*t*_2_	Waist thickness of hinge-2	2.2 mm
*d*	Width of mass block	10 mm
*h*	Height of mass block	30 mm
*w*	Width of hinges	8 mm
*l*	Fiber pasting span	5 mm
*A*_f_	Section area of optical fiber	1.227 × 10^−8^ m^2^
*E*_f_	Young’s modulus of optical fiber	70 GPa
*E*	Young’s modulus of 304 steel	210 GPa
*ρ*	Density of 304 steel	7850 kg·m^−3^
*μ*	Poisson’s ratio of 304 steel	0.3
g	Gravitational acceleration	9.8 m·s^−2^

**Table 2:** Summary of the characteristics of reported medium–highfrequency FBG accelerometers.

Ref	Resonance Frequency	Sensitivity	Fiber Type	Temperature Self-Compensation
Stefani [28]	3000 Hz	19 pm/g	Polymer FBG	No
Dai [29]	2918 Hz	13.82 pm/g	Silica FBG	No
Guo [30]	3600 Hz	1.7 pm/g	Metalized FBG	No
Wang [31]	3806 Hz	4.01 pm/g	Silica FBG	Yes
Wu [32]	8356 Hz	0.46 pm/g	Silica FBG	Yes
This article	2800 Hz	21.8 pm/g	Silica FBG	Yes

## Data Availability

Not applicable.
